# Mixed vulnerabilities: the biological risk of high parity is aggravated by emergency referral in Benin, Malawi, Tanzania and Uganda

**DOI:** 10.1186/s12939-025-02379-5

**Published:** 2025-01-20

**Authors:** Manuela Straneo, Lenka Beňová, Thomas van den Akker, Muzdalifat S. Abeid, Elizabeth Ayebare, Jean-Paul Dossou, Greta Handing, Bianca Kandeya, Andrea B. Pembe, Claudia Hanson

**Affiliations:** 1 Health Systems and Policy Global Public Health, Karolinska Institutet, Stockholm, Sweden; 2 Athena Institute, Vrije University, Amsterdam, The Netherlands; 3 Institute of Tropical Medicine, Antwerp, Belgium; 4 London School of Hygiene &Tropical Medicine, London, UK; 5 Department of Obstetrics and Gynecology, Leiden University Medical Centre, Leiden, The Netherlands; 6 Medical College East Africa, Aga Khan University, Dar Es Salaam, Tanzania; 7 Department of Nursing, College of Health Sciences, Makerere University, Kampala, Uganda; 8 Centre de Recherche en Reproduction Humaine Et en Démographie (CERRHUD), Cotonou, Benin; 9 Department of Student Affairs, Baylor College of Medicine, Houston, TX USA; 10 Center for Reproductive Health, Kamuzu University of Health Sciences, Private Bag 360, Chichiri BT3, Blantyre, Malawi; 11 Department of Obstetrics and Gynaecology, Muhimbili University of Health and Allied Sciences, Dar Es Salaam, United Republic of Tanzania

**Keywords:** Childbirth, Vulnerability, Hospitals, High parity, Emergency referral, Intrapartum care, Perinatal health, Maternal health, Fresh stillbirths, Very early neonatal mortality, Sub-Saharan Africa

## Abstract

Identification of interacting vulnerabilities is essential to reduce maternal and perinatal mortality in sub-Saharan Africa (SSA). High parity (≥ 5 previous births) is an underemphasized biological vulnerability linked to poverty and affecting a sizeable proportion of SSA births. Despite increased risk, high parity women rarely use hospitals for childbirth. We assessed whether emergency referral during childbirth was associated with adverse events in high parity women in hospitals in Benin, Malawi, Tanzania and Uganda. We used e-registry data collected in 16 hospitals included in the Action Leveraging Evidence to Reduce perinatal morbidity and morTality (ALERT) trial. Main outcomes were severe maternal outcomes and in-facility peripartum death (fresh stillbirth or very early neonatal death). Main exposure was parity; emergency (in-labour) referral was included as effect modifier with potential confounders. We used multivariable logistic regression including parity/referral interaction and post-regression margins analysis. Among 80,663 births, 4,742 (5.9%) were to high parity women. One third reached hospital following emergency referral. Severe maternal outcomes and peripartum mortality were over 2.5-fold higher in high parity women with emergency referral compared to the lowest risk group. To avert these adverse events, emergency referral must be avoided by ensuring high parity women give birth in hospitals.

**Trial registration** Pan African Clinical Trial Registry (www.pactr.org): PACTR202006793783148. Registered on 17th June 2020.

**Supplementary Information:**

The online version contains supplementary material available at 10.1186/s12939-025-02379-5.

## Background

Progress in reduction of maternal and perinatal mortality in sub-Saharan Africa (SSA) is slower than expected, despite increasing proportions of births taking place in health facilities and with skilled attendance [1]. In 2020, the highest maternal mortality ratio, at 545 deaths per 100,000 live births, was reported in SSA—corresponding to 202,000 deaths [2]. A total of 1.1 million neonatal deaths and 881,000 stillbirths were estimated to have happened in the region that same year [1].

Identification of interconnected clinical and socio-demographic vulnerabilities is crucial for implementing targeted strategies to improve maternal and perinatal outcomes [3]. One vulnerability that has received limited attention is high parity or grand multiparity (defined as ≥ 5 previous births)—affecting approximately one fifth of births in SSA [4, 5]. This biological risk factor is associated with adverse maternal and perinatal outcomes in low-resource though not in high-resource settings, probably due to very low prevalence and effective health care in the latter [6–13]. High parity is strongly correlated with sociological vulnerability (poverty) [14]. Our previous work has highlighted that high parity women are not sufficiently reached with health services. A large multi-country analysis found that a minority of women of high parity gave birth in hospitals in rural SSA (ranging between 1–23% across 18 countries) [5]. Furthermore, our in-depth analysis of Tanzania indicated that use of hospitals for childbirth among rural, poor, high parity women was very low (around 10%), despite antenatal indications on a hospital birth, with no improvement over the past 25 years [15, 16].

Limited emergency referral to hospitals, where advanced management of obstetric complications (such as blood transfusions and caesarean sections) is generally available, may constitute a barrier for high parity women to reach appropriate care when complications arise, adding to their vulnerability [3]. There is limited evidence on referral systems in SSA, and it represents a research priority [17]. Available evidence suggests weak or unreliable transport systems, with limited means of communication between facilities [18]. We thus hypothesized that emergency referral may compound the impact of the biological risk factor high parity, leading to worse outcomes in women and their babies.

We aim to fill the knowledge gaps around the impact of high parity on birth outcomes and the association with referral practices, to support policy and programming. The objective of this study was to assess perinatal mortality among high parity women who gave birth in 16 hospitals in Benin, Malawi, Tanzania and Uganda, and the extent to which referral modified these outcomes.

## Methods

### Setting

The study included data from women giving birth in 16 hospitals across four countries in SSA—Benin, Malawi, Tanzania and Uganda. As part of the Action Leveraging Evidence to Reduce perinatal morTality and morbidity (ALERT) trial [19], data were collected using an electronic registry (e-registry). This implementation study aimed to improve intrapartum care and reduce in-facility perinatal mortality with a multi-component intervention [20]. Selected characteristics of the countries are summarized in Table 1. Different types of hospitals were included in the study, comprising general district hospitals, referral hospitals, and one tertiary university hospital. Hospitals were mostly rural, public or private non-for-profit, representing the landscape of hospitals in SSA. There were ample differences in health system organization, staff cadres in childbirth care and fee structure across countries, which were detailed elsewhere [20, 21].

**Table 1:** Characteristics of countries with hospitals participating in the ALERT trial

	Population^ab^ (in million)	Births to high parity women out of all births^c^	Percentage of facility births out of all births^c^	Percentage of hospital births out of all births^c^
Benin	13.0	18%	85%	30%
Malawi	20.0	14%	94%	33%
Tanzania	65.6	20%	67%	32%
Uganda	45.9	23%	73%	36%

^a^In 2021, start of ALERT

^b^Data from http://data.worldbank.org

^c^Demographic and Health Survey (DHS) data [4]. Most recent DHS: Benin 2017, Malawi 2015, Tanzania 2015–16, Uganda 2016

### Population and inclusion criteria

We included women giving birth in these hospitals between July 1st, 2021, and December 30th, 2022. All births of babies of at least 28 weeks gestation or weighing ≥ 1000 g were included. Births before reaching hospital were excluded.

### Data collection

Data was collected by maternity ward staff or data clerks who were nurse-midwives by profession. Depending on in-country preference, data was abstracted from standardized paper-based records, including antenatal cards, clinical notes, and case notes on pregnancy risks, childbirth care and outcomes. The Research Electronic Data Capture (REDCap) [22] platform on tablets was used for data entry and storage. Details of the e-registry have been described [19].

### Variables

Two outcome variables were examined. Severe maternal outcomes was defined using pragmatic criteria [20], and included women who required major interventions (blood transfusion, laparotomy, hysterectomy, admission to higher level facility or to intensive care unit) and those who had died. The denominator for this outcome was women admitted for childbirth. The second was facility peripartum mortality defined as a baby classified as fresh stillbirth or born alive but died within 24 h of birth (very early neonatal death). Classification of fresh stillbirths was based on visual inspection by the staff attending the birth, as other definitions (e.g., stillbirth with foetal heart present on admission) were not reliable due to limited foetal monitoring capacity in the hospitals as reported in similar contexts [23]. To allow for inclusion of all births in the sample, for this outcome analysis was carried out per baby, thus the denominator was births among women admitted for childbirth.

Parity (nulliparity, 1–4, ≥ 5) was the main exposure variable. High parity women were those with five or more previous births. Referral status was the effect modifier, with three categories: no referral, woman who arrived at one of the hospitals from home or other facilities following referral during pregnancy (pre-labour referral) or after the start of labour (emergency referral).

As potential confounders, we considered woman’s age group at index birth (< 19, 20–29, 30–39, 40–49 years), number of antenatal care (ANC) visits during index pregnancy (none, 1–3, ≥ 4), presence or absence of any antenatal risk factor (multiple pregnancy, previous caesarean section (CS), any hypertensive disorder, diabetes/gestational diabetes, any other medical condition), presence or absence of intrapartum risk factors (malpresentation, suspected small for gestational age, post-term, chorioamnionitis, antepartum haemorrhage, pre-term labour), birth weight group (1000–1999, 2000–2999, 3000–3999, ≥ 4000 g), country and use of uterotonics during labour.

### Statistical analysis

Analysis was carried out using STATA IC version 16. Characteristics of women per country who had given birth in the study period were described as percentages with 95% confidence intervals (CI). Successive analysis was carried out on pooled data for all countries were analysed to maximise the sample size due to both outcomes being relatively rare. Analysis of the outcome variables by parity, referral and potential confounders was carried out using percentages and confidence intervals.

Births by woman’s referral status stratified by potential confounders were calculated as percentages, with 95% CI. In bivariate analysis, we computed the percentages of outcomes by women’s and births’ characteristics, with 95% CI and *p*-values from chi-squared tests.

Odds ratios (OR) of the outcomes were calculated using logistic regression, accounting for survey effect and hospital-level clustering. We included referral status (not referred, referred pre-labour, or following emergency referral) as an effect modifier by using an interaction term in the multivariable model. *P*-values < 0.05 were considered significant. All confounders significant in bivariate logistic regression were included in the final model. We tested whether the logistic regression model with the interaction term compared to the one without interaction was a better fit using the likelihood-ratio test. Using post-estimation margins analysis following on from the final adjusted model, we computed the absolute risk of a maternal severe outcome or of a peripartum death (with a 95% CI) for the nine combinations of parity and referral status.

## Results

We included 78,085 women (Table S2). All variables used had low (< 1.5%) levels of missingness. The overall percentage of women of high parity in the sample was 5.8%, ranging from 3.1% in Tanzania to 8.9% in Benin. Approximately one quarter of women (26.8%) in the pooled sample had reached hospitals following emergency referral, with wide variation across countries (from 5.2% in Tanzania to 47.2% in Benin). Only 2.9% were referred in-pregnancy (range 1.2% in Uganda to 7.3% in Benin). Hospitals in Benin recorded a higher percentage of women with risk factors, both antenatal (45.6%) and intrapartum (28.0%), while percentages of women with risk factors were lowest in Malawi (9.0% antenatal, 4.7% intrapartum). CS rates were highest in hospitals in Benin (45.5%) and lowest in Malawi (17.3%).

There were 4,742 births to high parity women. Among them, one third (33.4%) followed emergency referral. The lowest percentage of emergency referral was among babies born to women of parity 1–4 (24.2%) (Table S3). The percentage of babies born following maternal emergency referral varied markedly across countries, from 5.3% (Tanzania) to 46.3% (Benin). Pre-labour referrals were uncommon, ranging from 1.2% in Uganda to 7.1% in Benin among births to all women regardless of parity. The percentage of women who gave birth in hospitals without referral ranged from 46.2% in Benin to 93.4% in Tanzania.

### Severe maternal outcomes

Among 78,085 women, there were 2,209 (2.8%, 95% CI, 1.9–4.2) severe maternal outcomes (Table 2). Severe outcomes were more frequent among high parity ≥ 5 women (5.5%) compared to other parity groups. They were more common in women who reached hospitals following in pregnancy referral (10.8%, compared to 4.2% in emergency referral), though confidence intervals are wide due to small sample size in the former. Across the four countries, severe outcomes were observed more commonly in Benin (5.8%) compared to other countries.

**Table 2:** Risk of severe maternal outcomes among women (*n* = 78,085) and perinatal death among births (*n* = 80,663) by maternal and obstetric characteristics in 16 ALERT hospitals in Benin, Malawi, Tanzania and Uganda, between July 1st, 2021, and December 31st, 2022

	Severe maternal outcomes				Perinatal deaths			
Variable	Number of women	%	95% CI	*p*-value*	Number of babies	%	95% CI	*p*-value*
**Total**	**2,209**	2.8	1.9–4.2		**2,323**	2.9	1.9–4.3	
**Parity (*n* = 78,085)**					**(*n* = 80,663)**			
0	731	2.3	1.6–3.5		755	2.4	1.7–3.3	
1–4	1,233	2.9	2.0–4.2		1,285	2.9	1.9–4.5	
≥ 5	245	5.5	3.7–7.9	< 0.001	283	5.9	4.1–8.5	< 0.001
**Referral status (*n* = 77,789)**					**(*n* = 80,370)**			
Not referred	1086	2.0	1.6–2.5		986	1.7	1.3–2.4	
Pre-labour	244	10.8	5.7–19.6		137	5.8	3.4–9.7	
Emergency	872	4.2	2.5–6.8	< 0.001	1,192	5.5	3.4–8.9	< 0.001
**Woman’s age at time of birth (*n* = 77,978)**					**(*n* = 80,558)**			
10–19	33	2.0	1.4–2.7		394	2.3	1.5–3.4	
20–29	1,119	2.8	1.8–4.1		1,128	2.7	1.8–4.0	
30–39	655	3.6	2.4–5.2		719	3.7	2.5–5.5	
40–49	100	5.0	3.2–7.7	< 0.001	80	3.8	2.4–6.1	< 0.001
**Country (*n* = 78,085)**					**(*n* = 80,663)**			
Benin	933	5.8	4.0–8.4		892	5.2	3.5–7.5	
Malawi	412	1.5	1.1–2.0		447	1.6	1.3–2.0	
Tanzania	304	2.3	1.5–3.5		143	1.1	0.6–1.8	
Uganda	560	2.6	1.5–4.2	< 0.001	841	3.7	2.6–5.3	< 0.001
**ANC visits (*n* = 77,086)**					**(*n* = 79,817)**			
** 0**	18	2.1	1.0–4.4		40	4.6	3.1–6.8	
1–3	898	3.2	2.0–5.1		1,091	3.8	2.4–5.9	
≥ 4	1,233	2.5	1.7–3.7	< 0.001	1,151	2.3	1.5–3.5	< 0.001
**Any antenatal risk factor (*n* = 78,085)**					**(*n* = 80,663)**			
Not present	1,369	2.2	1.5–3.1		1,642	2.6	1.7–3.9	
Present	840	3.8	3.8–7.3	< 0.001	681	4.1	3.1–5.3	< 0.001
**Any admission risk factors (*n* = 78,085)**					**(*n* = 80,663)**			
Not present	1,361	2.0	1.5–2.6		1,472	2.1	1.4–3.0	
Present	848	9.1	6.4–12.8	< 0.001	851	8.1	6.4–10.2	< 0.001
**Mode of birth (*n* = 77,834)**					**(*n* = 80,658)**			
Spontaneous vaginal birth	909	1.6	1.2–2.3		1,322	2.3	1.5–3.7	
Caesarean section	1,206	5.6	4.0–7.8		917	4.1	3.0–5.5	
Assisted vaginal birth (vacuum/forceps)	26	5.8	4.1–8.0		28	6.1	4.0–9.0	
Breech	28	5.3	3.7–7.6	< 0.001	56	8.5	6.7–10.7	< 0.001
**Birth weight (grams) (*n* = 77,472)**					(***n* = 80,236)**			
1000–1999	332	9.9	6.2–15.4		463	11.4	9.6–13.4	
2000–2999	917	2.9	1.8–4.4		915	2.7	1.6–4.5	
3000–3999	827	2.1	1.5–2.9		821	2.0	1.4–3.0	
≥ 4000	64	2.8	1.8–4.4	< 0.001	61	2.7	1.9–3.8	< 0.001
**Use of uterotonics (*n* = 78,014)**					**(*n* = 80,636)**			
No	916	1.7	1.3–2.3		979	1.8	1.4–2.4	
Yes	1,289	5.1	3.3–7.7	< 0.001	1,343	5.0	3.8–6.5	< 0.001

^*^based on chi-squared

### Peripartum deaths

Among 80,663 babies in the sample, there were 2,323 peripartum deaths (1,706 fresh stillbirths and 617 very early neonatal deaths) corresponding to an overall peripartum mortality rate of 2.9% (95% CI, 1.9–4.3) (Table 2), ranging from 1.1% in Tanzania to 5.2% in Benin. Peripartum deaths were more frequent among babies born to high parity women (5.9%), compared to lower parity women (2.4% in nulliparous women and 2.9% in women at parity 1–4). Deaths were more frequent among women with emergency (5.5%) or pre-labour (5.8%) referral compared to women not referred (1.7%).

### Logistic regression

#### Severe maternal outcome

**Fig. 1:**
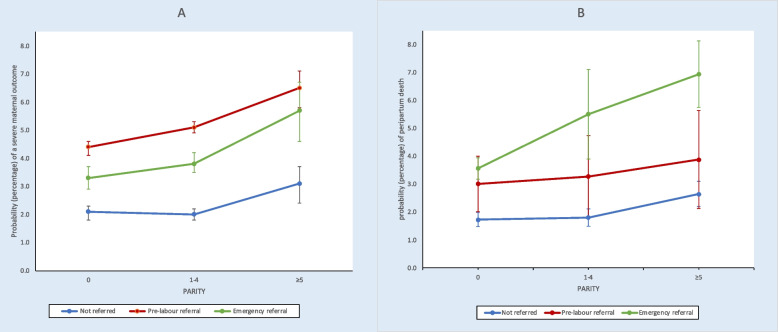
A: Estimated probability, with 95% CI, of a severe maternal outcome among women giving birth, and B: Estimated probability, with 95% CI, of peripartum death among infants born, by combination of woman’s parity and referral status, in hospitals in Benin, Malawi, Tanzania, Uganda, using post-estimation margins analysis

In crude analysis, all variables were significantly associated with this outcome. In adjusted analysis (Table 3), women of high parity has increased odds of a severe maternal outcome compared to women of intermediate parity (OR 1.54, 95% CI, 1.25–1.89). Women who were referred had higher adjusted odds of a severe maternal outcome compared to non-referred women: 2.49 (95% CI, 1.86–3.34) for pre-labour referral and 1.86 (95% CI, 1.49–2.31) for emergency referral. For this outcome, despite the likelihood ratio test was non-significant (*p* = 0.51) for the fit of the model with interaction, for consistency with the peripartum outcome, to estimate the probability of a severe maternal outcome for combinations of parity and referral, post-estimation margins analysis was performed following logistic regression including an interaction term (Fig. 1-A and Table S6-A). Women of high parity reaching hospitals following emergency referral had a 5.7% (95% CI, 4.6–6.7) probability of a severe maternal outcome, and for those who reached hospitals following in pregnancy referral it was 6.5% (95% CI, 3.8–9.2). By contrast, in the lower risk group (nulliparous women with no referral), the risk was 2.0% (95% CI, 1.8–2.3).

**Table 3:**
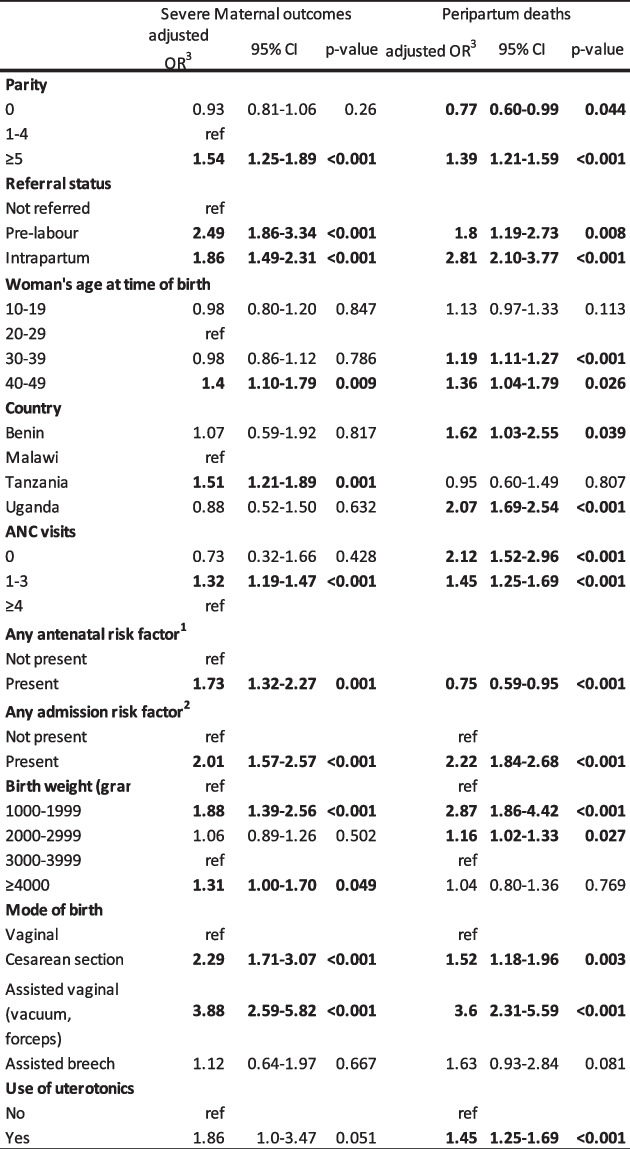
Adjusted odds ratios of a severe maternal outcome among women giving birth (*N*=76,291) (left) and of a peripartum death among babies (*N*=79,062) (right) born in hospitals included in the ALERT trial in Benin, Malawi, Tanzania, Uganda

^1^multiple pregnancy, previous CS, hypertension, diabetes/gestational diabetes, premature rupture of membranes, HIV positivity, VDRL positivity, anaemia, cardiac disease, malaria

^2^Malpresentation, preterm labour, post term, antepartum haemorrhage, small for gestational age, chorioamnionitis

^3^Adjusted for parity, referral status, maternal age at birth, country, ANC visits, presence of any antenatal risk factor, presence of any admission risk factor, birth weight, mode of birth, use of oxytocics

#### Peripartum death

In the crude logistic regression model, all variables examined were significantly associated with peripartum death. In the adjusted model (Table 3), babies born to women of high parity had 1.39 (95% CI, 1.21–1.59) higher adjusted odds of peripartum death compared to women of parity 1–4, while those of nulliparous women had reduced odds of a perinatal death (OR 0.77, 95% CI 0.60–0.99). Babies born to women who reached hospitals following referral had higher adjusted odds of a peripartum death; for those referred pre-labour the adjusted odds ratio was 1.80 (95%CI 1.19–2.73), while for women referred intrapartum it was 2.81 (95% CI, 2.10–3.77). We also found that the adjusted odds of peripartum death were higher in Benin (1.62, 95% CI, 1.03–2.55) and Uganda (2.07, 95% CI, 1.69–2.54) compared to Malawi.

For peripartum deaths, the likelihood-ratio test indicated the model with interaction was a better fit than that without interaction (*p* = 0.0006). Using margins analysis following the final logistic model with interaction, we found that the probability of peripartum death was highest among women of high parity who had been referred intrapartum—6.9% (95% CI 5.7–8.1). In comparison, babies born to high parity women who were not referred had a probability of peripartum death of 2.6% (Fig. 1-B and Table S6-B). For high parity women who were referred pre-labour, the probability was 3.9% (95% CI, 5.7–8.1). Babies of nulliparous women who had not been referred had the lowest risk of peripartum death, 1.7% (95% CI 1.5–2.0).

## Discussion

Our analysis of cross-sectional data of 78,085 women giving birth to 80,663 babies across hospitals in Benin, Malawi, Tanzania, and Uganda suggests that referral is not effective in preventing peripartum mortality and adverse maternal outcomes, and inequitably affected babies of high parity women. The combination of the biological vulnerability of high parity and of emergency obstetric referral resulted in substantially higher severe maternal outcomes and peripartum mortality.

Firstly, our key finding is that the effect of the double disadvantage of high parity and emergency referral is leading to a 2.5-fold increase of severe maternal outcomes and peripartum mortality compared to the lowest risk group (nulliparous, no referral). To our knowledge, no studies have examined this double vulnerability, but several studies indicate that high parity is a risk factor in this context [9–13] and referral itself is associated with increased risk [24–27]. Complications arising in labour require rapid action to avoid maternal and foetal repercussions. The referral system is the backbone on which the health system relies to save women and babies. If referral works well, then even among referred women, peripartum mortality should be low. Our findings indicate that emergency referral substantially impacted outcomes negatively. This is in line with findings that proximity to facilities with higher obstetric capability reduced intrapartum stillbirths in Ghana [28] and direct maternal mortality in Tanzania [29]. Limited geographical accessibility to hospitals in SSA has been described [30] in urban and rural settings [31–33], resulting in long and uncertain travel times. Our analysis hints to a greater effect on peripartum mortality compared to maternal outcomes. Complications arising during birth will put babies at risk before the woman. Antepartum haemorrhage for instance, may compromise blood flow to the foetus, resulting in a hypoxic insult before the mother is a risk of a severe outcome. The greater vulnerability of babies is compounded by diagnostic challenges of foetal distress in this context. To avert maternal deaths, a two hour travel time to facilities that can manage childbirth complications is currently recommended [34]. This travel time may be too long to save babies once complications arise; in high income settings, a travel time of 20 min is suggested to avert foetal adverse outcomes [35].

Secondly, our study adds to previous evidence on the vulnerability of high parity [6, 9–13, 36, 37]. High parity is an important biological risk [3], particularly among rural and poor women, compounded by their low use of hospitals for childbirth [5, 15, 16]. In this study, only 6% of births in the hospitals were to high parity women, while population data shows that the percentage of births to women of parity ≥ 5 ranges between 14% (Malawi) to 23% (Uganda) [4]. Women of high parity are advised to give birth in a higher-level facility – mostly in hospitals – where complications can be readily addressed [38, 39]. There are multiple reasons for delays in reaching hospitals for women of high parity. It is likely that they result from the interacting effect of high parity, many household responsabilities, poverty, low education and power, in addition to a likely low risk perception [16]. Our hospital-based data indicates that these women reached hospitals following referral, but we don’t know why they are arriving late. There are pointers from the literature (including money, empowerment, distance, knowledge) [25, 40, 41], but we see this happening in all for countries and call for context-specific identification of barriers/facilitators to improving in-pregnancy as well as in-labour referral.

The question that emerges from these findings is which reparative strategies are possible in this context to reduce the disadvantage in terms of severe maternal outcomes and peripartum mortality? High parity as a biological factor is tightly linked to poverty [42, 43]. Barriers to these women’s use of hospitals result from the complex interaction of high parity, low socio-economic status and women’s low autonomy and power. To avoid the need for referral during labour, research is needed to identify and evaluate strategies to improve access for high parity women to hospital childbirth care. These may include financial aid for transport or maternity waiting homes [5]. Though policies recommending childbirth care in hospital exist in these countries [38, 39, 44], women’s limited adherence with referral advice [45, 46] has been described. Further studies can help elucidate whether obstetric risk is adequately recognized by health care workers and in communities.

Based on these concerning findings of the impact of emergency referral on adverse outcomes for high parity women and their babies, we echo calls made by others [47, 48] on the need for policy focus on women at higher risk. The contextually specific solution in the present scenario is in-pregnancy referral to hospitals for childbirth in higher risk women (such as those of high parity) where emergency referral is unreliable. Policy measures are necessary to overcome barriers to use of hospitals to give birth for these higher-risk women.

### Strength and limitations

The main strength of this study is the large size of the sample. Additional strengths are the multi-country design and high-quality of data, the novel perspective on women at high parity, and its association with referral status. Limitations should be considered in interpreting the findings. The hospitals included in the ALERT trial, though typical in the settings, are not representative of the countries, thus, findings cannot be generalized. Since socio-economic and other women’s characteristics (such as marital status, education, distance to hospital) were not included in the e-registry data, the outcomes could not be adjusted for these variables.

As the data was collected in hospitals, there was no available information on neonatal survival past the first 24 h of life. Though the definition of a fresh stillbirth was based on visual inspection of the foetus by the staff assisting the birth – due to limited capacity of foetal monitoring – and may have caused some misclassification of stillbirths, this is unlikely to be a major source of bias given the size of the effect. Though in some countries women with an intrauterine foetal death are referred to hospital for childbirth, this is unlikely to have an effect on the risk of mortality observed, as these babies would be classified as macerated stillbirths, which were excluded from analysis. Due to the relatively low frequency of the outcome, we could not analyse outcomes by country. Thus, a more detailed, context-specific analysis could not be carried out. We were limited by the nature of the data and the selection of the women in the sample in our ability to delve into the possible explanations underlying our findings. For example, the limited number of women referred in pregnancy did not allow to examine this group in more detail, such as disaggregating for key obstetric complications.

## Conclusion

This analysis of 78,085 women giving birth to more than 80,000 babies found that women of high parity who reached hospitals for childbirth following intrapartum referral in Benin, Malawi, Tanzania, and Uganda had a substantially higher risk both of a severe maternal outcome and of peripartum mortality. The findings suggest that the mixed vulnerabilities resulting from the interaction of biological and sociological threats and delays during the obstetric referral process have substantial effects on outcomes of the mother-baby dyad.

To reduce the need for intrapartum referral, measures are urgently needed to improve high parity women’s use of hospitals. These may include financial aid to overcome economic barriers and maternity waiting homes, as well as greater attention on this risk factor during ANC to support hospital births.

## Supplementary Information

Supplementary Material 1.

Supplementary Material 2.

Supplementary Material 3.

## Data Availability

The data analysed will be made publicly available after the finalisation of the trial, as outlined in the data management plan and publication policy. Before 2027, the data is available on request.

## Abbreviations

ALERT: Action Leveraging Evidence to Reduce perinatal Mortality and morbidity trial
ANC: Antenatal care
CS: Caesarean section
DHS: Demographic and Health Survey
OR: Odds ratio
REDCap: Research Electronic Data Capture
SSA: Sub-Saharan Africa
