# Supplementary material for: Mixed vulnerabilities: the biological risk of high parity is aggravated by emergency referral in Benin, Malawi, Tanzania and Uganda
Source: Int J Equity Health. 2025 Jan 20;24:19. doi: 10.1186/s12939-025-02379-5 (PMC11744807; doi:10.1186/s12939-025-02379-5)
Supplement: Supplementary file 1 — Supplementary Material 1. [file 12939_2025_2379_MOESM1_ESM.docx]

**Table 2 (Supplementary)** – Characteristics of 78,085 women who had given birth in hospitals in Benin, Malawi, Tanzania and Uganda, part of the ALERT study (July 1^st^, 2021 to December 31st, 2022), overall and by country
