# Supplementary material for: Mixed vulnerabilities: the biological risk of high parity is aggravated by emergency referral in Benin, Malawi, Tanzania and Uganda
Source: Int J Equity Health. 2025 Jan 20;24:19. doi: 10.1186/s12939-025-02379-5 (PMC11744807; doi:10.1186/s12939-025-02379-5)
Supplement: Supplementary file 2 — Supplementary Material 2. [file 12939_2025_2379_MOESM2_ESM.docx]

**Supplementary Table 2** – Babies (numbers, row percentages with 95% CI) by woman’s referral status born in hospitals in Benin, Malawi, Tanzania and Uganda included in the ALERT trial, between July 1st, 2021 and December 31st, 2022.

|  | Not referred | | | Pre-labour referral | | | Intra-partum referral | | |  |
| --- | --- | --- | --- | --- | --- | --- | --- | --- | --- | --- |
| Variable | N. | % of total | 95% CI | N. | % of total | 95% CI | N. | % of total | 95% CI | p-value^1^ |
| **Parity (n=80,370)** | |  |  |  |  |  |  |  |  |  |
| 0 | 21185 | 67.2 | 53.0-78.9 | 991 | 3.1 | 1.6-6.0 | 9329 | 29.6 | 19.1-42.8 |  |
| 1-4 | 32259 | 73.1 | 59.9-83.2 | 1181 | 2.7 | 1.5-4.7 | 10683 | 24.2 | 14.9-36.9 |  |
| ≥5 | 2972 | 62.7 | 45.3-77.3 | 186 | 3.9 | 2.2-7.0 | 1584 | 33.4 | 19.6-50.8 | <0.001 |
| **Woman’s age (n=80,265)** | | |  |  |  |  |  |  |  |  |
| 10-19 | 11611 | 67.9 | 52.3-80.3 | 457 | 2.7 | 1.2-6.0 | 5034 | 29.4 | 18.3-43.8 |  |
| 20-29 | 29334 | 70.1 | 56.5-80.9 | 1168 | 2.8 | 1.5-5.0 | 11350 | 27.1 | 17.2-40.0 |  |
| 30-39 | 13839 | 72.0 | 58.2-82.5 | 662 | 3.4 | 1.9-6.0 | 4733 | 24.6 | 14.9-37.9 |  |
| 40-49 | 1584 | 76.3 | 11.7-33.8 | 65 | 3.1 | 1.6-6.0 | 428 | 20.6 | 11.7-33.8 | <0.001 |
| **Country (n=80,370)** | |  |  |  |  |  |  |  |  |  |
| Benin | 8046 | 46.2 | 35.6-57.9 | 1233 | 7.1 | 3.8-13.1 | 7994 | 46.3 | 32.1-61.1 |  |
| Malawi | 18232 | 66.3 | 41.9-84.4 | 689 | 2.5 | 0.7-8.3 | 8558 | 31.1 | 14.6-54.5 |  |
| Tanzania | 12496 | 93.4 | 78.7-98.2 | 178 | 1.3 | 0.2-7.9 | 704 | 5.3 | 1.7-15.3 |  |
| Uganda | 17642 | 79.3 | 74.4-83.5 | 4340 | 1.2 | 0.5-2.5 | 4340 | 19.5 | 15.5-24.2 | <0.001 |
| **ANC visits (n=79,537)** | |  |  |  |  |  |  |  |  |  |
| 0 | 518 | 59.6 | 38.1-78.0 | 10 | 1.2 | 0.5-2.7 | 341 | 39.2 | 21.2-60.8 |  |
| 1-3 | 19469 | 68.1 | 53.1-80.0 | 878 | 3.1 | 1.6-5.8 | 8255 | 28.9 | 17.8-43.2 |  |
| 4+ | 36073 | 72.1 | 57.7-83.0 | 1411 | 2.8 | 1.5-5.1 | 12582 | 25.1 | 15.2-38.6 | <0.001 |
| **Any antenatal risk factor^2^ (n=80,370)** | | | |  |  |  |  |  |  |  |
| Not present | 40431 | 72.2 | 57.3-83.4 | 885 | 1.6 | 0.7-3.3 | 14713 | 26.3 | 15.7-40.5 |  |
| Present | 15985 | 65.7 | 53.7-75.9 | 1473 | 6.1 | 3.7-9.7 | 6883 | 28.3 | 18,9.40.0 | <0.001 |
| **Any admission risk factor^3^ (n=80,370)** | | | |  |  |  |  |  |  |  |
| Not present | 45911 | 72.1 | 58.6-82.5 | 1333 | 2.1 | 1.1-4.0 | 16429 | 25.8 | 16.1-38.6 |  |
| Present | 10505 | 62.9 | 47.5-76.0 | 1025 | 6.1 | 3.4-10.9 | 5167 | 30.9 | 19.3-45.7 | <0.001 |

^1^using chi-squared test

| ^2^ multiple pregnancy, previous CS, hypertension, diabetes/gestational diabetes, premature rupture of membranes, HIV positivity, VDRL positivity, anemia, cardiac disease, malaria | |
| --- | --- |
| ^3^ Malpresentation, preterm labour, post term, antepartum haemorrhage, small for gestational age, chorioamnionitis |  |
