# Supplementary material for: Mixed vulnerabilities: the biological risk of high parity is aggravated by emergency referral in Benin, Malawi, Tanzania and Uganda
Source: Int J Equity Health. 2025 Jan 20;24:19. doi: 10.1186/s12939-025-02379-5 (PMC11744807; doi:10.1186/s12939-025-02379-5)
Supplement: Supplementary file 3 — Supplementary Material 3. [file 12939_2025_2379_MOESM3_ESM.docx]

**Supplementary Table 4 –** Probability of a perinatal death by parity and referral status in babies born in Benin, Malawi, Tanzania, Uganda by post-estimation margins analysis (N=79063)

| Parity | Referral status | Probability of a perinatal death | 95% CI | p-value |
| --- | --- | --- | --- | --- |
| 0 | not referred | 1.7 | 1.5-2.0 | <0.001 |
| 0 | referred pre-labour | 3.0 | 2.0-4.0 | <0.001 |
| 0 | referred in-labour | 3.6 | 3.2-3.9 | <0.001 |
| 1-4 | not referred | 1.8 | 1.5-2.1 | <0.001 |
| 1-4 | referred pre-labour | 3.3 | 1.8-4.7 | <0.001 |
| 1-4 | referred in-labour | 5.5 | 3.9-7.1 | <0.001 |
| ≥5 | not referred | 2.6 | 2.2-3.1 | <0.001 |
| ≥5 | referred pre-labour | 3.9 | 2.1-5.6 | <0.001 |
| ≥5 | referred in-labour | 6.9 | 5.7-8.1 | <0.001 |
